# 

**Published:** 1850-05

**Authors:** 


### NOTICE TO CORRESPONDENTS.

Communications and Books for notice should be addressed to the Editor, care of Messrs. Lindsay & Blakiston.

Letters, &c., connected with the *business affairs* of the Journal should be addressed to the Publishers.

Papers for publication must be received *before* the 20th of the month, or they cannot appear in the forthcoming number.

Several of our domestic exchanges have either come very late to hand, or have entirely failed to reach us. Those only that have been received since April 1st are included in the following list.

The Boston Medical and Surgical Journal. (Weekly, Boston.)
Buffalo Journal.
New York Journal of Medicine.
Medical News.
Western Lancet.
The British American Journal of Medical and Physical Science. (Montreal.)
The London Lancet. (Weekly, London.)
The Medical Times. (Weekly, London.)
Dublin Medical Press.
Provincial Medical and Surgical Journal.
New Jersey Medical Reporter.
Transylvania Medical Journal.
Southern Medical and Surgical Journal.
Ohio Medical and Surgical Journal.
New Orleans Medical and Surgical Journal.

---

The following works have also been received for notice:

The Diseases of the Interior Valley of North America. By D. Drake, M. D., &c.

Remarks on Cholera. By E. J. Coxe, M. D., New Orleans.

Accommodation of the eye to distance. By W. C. Wallace, M. D. From the Author.

A Memoir on Stricture of the Urethra. By J. P. Mettauer, M. D., LL. D. From the Author.

Bennett on the Uterus. From Messrs. Lea & Blanchard.

Essays on the Puerperal Fever, by F. Churchill, M. D. From Messrs. Lea & Blanchard.

---

Communications have been received from:—

W. J. Reese, M. D., Alabama.
William Waters, M. D., Fredericktown, Md.

---

The foreign correspondents of the Examiner will please direct their Exchanges and other communications to the care of Mr. Charles J. Skeet, 27 King William St., Charing Cross, London, or Mr. H. Bossange, 21 Bis, Quai Voltaire, Paris.

